# Biodegradation effects of o-cresol by *Pseudomonas monteilii* SHY on mustard seed germination

**DOI:** 10.6026/97320630014271

**Published:** 2018-06-30

**Authors:** Shainy Nhattuketty Krishnan, Anuraj Nayarisseri, Usha Rajamanickam

**Affiliations:** 1 Department of Microbiology, Karpagam academy of higher education, Eachinary, Coimbatore- 641 021; 2 Department of Microbiology, Safi center for scientific research, Vazhayoor East, Malappuram - 673 633; 3 In silico Research Laboratory, Eminent Biosciences, Vijaynagar, Indore - 452010, Madhya Pradesh, India; 4 Bioinformatics Research Laboratory, LeGene Biosciences (P) Ltd, Indore - 452010, Madhya Pradesh, India

**Keywords:** *Pseudomonas monteilii* SHY, germination inhibition, mustard, seeds, *Brassica juncea*, bioremediation

## Abstract

Cresols are ubiquitous due to industrial production and natural presence. o-cresol (2-methyl phenol) is highly toxic to both fauna and flora. It has been included in the EPA list as one of the priority pollutants. The deleterious effects of pesticides, herbicides, and many other chemical compounds on seed germination are known. However, the effect of o-cresol on seed germination is not known. Therefore, it is of interest to study the effect of o-cresol on germination of 13 different vegetable crop seeds using standard Filter Paper Method. There is no effect on germination for brinjal, red chili, and (green gram, chickpea, cucumber, tomato, fenugreek, cowpea, Green pea, coriander, and spinach, seeds even at 1500 mg/l of o-cresol However, okra and mustard were found to be sensitive to ocresol. Germination of mustard under controlled concentration of o-cresol showed similar results by soil method. It was found that germination percentage and seedling vigour (Vigour Index) was reduced by o-cresol. The percent germination was reduced to 64 and 12 at 25 and 50 mg o-cresol/kg soil as against 100% in the case of untreated control. The vigour index was reduced to 160 and 10, respectively as against of 646 that for the control. The viability of seeds by 2,3,5 - tetrazolium trichloride (TTC) test showed that a considerable reduction was observed at 200mg/l o-cresol. Reduced protease and amylase activity in o-cresol shows inhibited mustard generation. However, mustard generation inhibition was restored by the bioremediation of o-cresol using Pseudomonas monteilii SHY. Thus, the biodegradation effects of o-cresol by Pseudomonas monteilii SHY on mustard seed germination are shown.

## Background

Cresols are the largest group of phenolic compounds occurring naturally in plants and as natural components of crude oil, coaltar and brown cresylic type mixtures. United States Environmental Protection Agency (USEPA) has classified all methyl derivatives of phenol (cresol isomers) as stable, priority chemical pollutants [1]. Cresol are present in ground water pollutants which occur in the environment through improper disposal and accidental spillage of effluents from industries such as pesticides, oil refining, coal gasification, dye manufacturing, petroleum refineries, and petrochemicals, pharmaceutical and resin manufacturing plants and due to their high stability, high toxicity and carcinogenicity they cause considerable damage and threat to the ecosystem [2, 3, 4]. Cresols are also released to soil at landfills and hazardous waste sites and even from poultry manure [5, 6, 7].

Germination was defined as "the emergence and development of the seedling to a stage where the aspect of its essential structures indicates whether it is able to develop further into a satisfactory plant under favorable conditions" (ISTA, 1985). Seed germination depends on many factors like temperature, water, oxygen and sometimes light and darkness. Seed dormancy prevents germination of viable seed for a certain period of time despite of favorable environmental conditions and dormancy is broken, changing the balance of growth inhibitors to growth promoters. A wide range of dormancy mechanisms has evolved to maximize the seed survival [8]. Respiratory inhibitors interfere with metabolic pathways and inhibit seed germination [9]. Amrutha (2014, unpublished data) has shown the inhibitory effect of phenol on the germination of seeds of chickpea, mung bean (green gram), and long-podded cowpea. Increased phytotoxicity of o-cresol on seven Chinese vegetables has been reported [10].

The above reports point to the fact that the cresols could be inhibitory to seed germination of crop plants. Being industrially important and widely used chemicals, phenol and cresols and their derivatives are likely to be present in the high concentrations in industrial wastewaters. They may cause severe pollution problems if disposed untreated. Hence, there is a need to eliminate these compounds from soil. Therefore, it is of interest to study the bioremediation effect of o-cresol on mustard seed germination using Pseudomonas monteilii SHY.

## Methodology

### Germination studies: O-cresol sensitivity assay by filter paper method

#### O-cresol sensitivity assay by filter paper method

Seeds of brinjal, (Solanum melongena), red chili, (Capsicum annuum), green gram, (Vigna radiata), chickpea, (Cicer arietinum), cucumber, (Cucumis sativus), tomato, (Solanum lycopersicum) fenugreek, (Trigonella foenum-graecum), cowpea, (Vigna unguiculata), Green pea, (Pisum sativum), coriander Coriandrum sativum), okra (Abelmoschus esculentus), mustard (Brassica juncea) and spinach, (Spinacia oleracea) were subjected to a preliminary screening for their sensitivity to different concentrations of ocresol (100, 500, 800, 1000, 1200 and 1500 mg/l) during germination by the standard Filter Paper Method (ISTA1985). Three replicates of 25 seeds and controls using distilled water were maintained for each concentration of the chemical.

### Secondary screening for sensitivity in soil

Seeds that were found to be sensitive to o-cresol by filter paper method were taken for a secondary screening in soil. Different concentrations of o-cresol (0, 25, 50, 100, 150 and 200 mg/kg) were added to the soil and moisture was maintained at 20%. Three replicates and controls were maintained for each variable. The germination percentage, shoot length, root length and vigour index were evaluated after 7 days [11].

### Seed viability by TTC test

Viability of the o-cresol treated and untreated seeds were tested using 2,3,5-tetrazolium trichloride (TTC) by the standard procedure of ISTA Rules (ISTA, 2009) [12]. The optical density of the extracted red color (formazan) was determined at 480 nm using Shimadzu UV-visible spectrophotometer.

### Protease assay and Amylase assay

Method of Laskowsky (1955) [13] was followed for the assay of protease activity. The enzyme activity was measured at 660nm in a Shimadzu spectrophotometer within 20 minutes. Amylase activity was assayed by measuring the release of reducing sugar from gelatinized soluble starch. (Bernfeld, 1955) [14]. Absorbance was measured at 540nm in a Shimadzu spectrophotometer and the values of A540 was expressed as the enzyme activity.

### *Pseudomonas monteilii* SHY inoculated soil

*P. monteilii* SHY isolated earlier in the laboratory was tested for bioremediation of o-cresol spiked soil. Soil spiked with 0, 25 and 50 mg o-cresol/kg soil was inoculated with the bacterial cells at an inoculum size of 1.6 x 108 CFU/gm. soil. Mustard seeds were sown immediately after inoculation of the soil. Restoration of normal germination of mustard seeds in the o-cresol-spiked soil and recovery of full vigour of the seedlings on application of the bacterial inoculum were taken as the criteria for evaluating its degradation efficiency under soil conditions.

### *Pseudomonas monteilii* SHY growth in soil

To determine the viability and the growth of the inoculated bacterial cells, standard plate count method was performed and the colonies were counted using a colony counter and the growth was expressed as Colony Forming Units (CFU) [11].

### Estimation of o-cresol in soil

1gm soil was collected every 24 h from the experimental cups spiked with different concentrations of o-cresol and the residual o-cresol present in soil were estimated using 4-aminoantipyrene method (Lacoste R. J. et al. 1959) [15].

### o-cresol degradation and seed germination

The effect of bacterial amendment of soil spiked with different concentration of o-cresol was estimated by calculating the germination percentage, and the root and shoot lengths of the seedlings after 8 days of sowing in all cases. The vigour index (VI) was calculated as (mean root length + mean shoot length) x percentage of germination [16].

## Results & Discussion

### Screening of Different Crop Seeds for Sensitivity to o-Cresol:Filter Paper Method

#### Filter Paper Method

**Figure 1:**
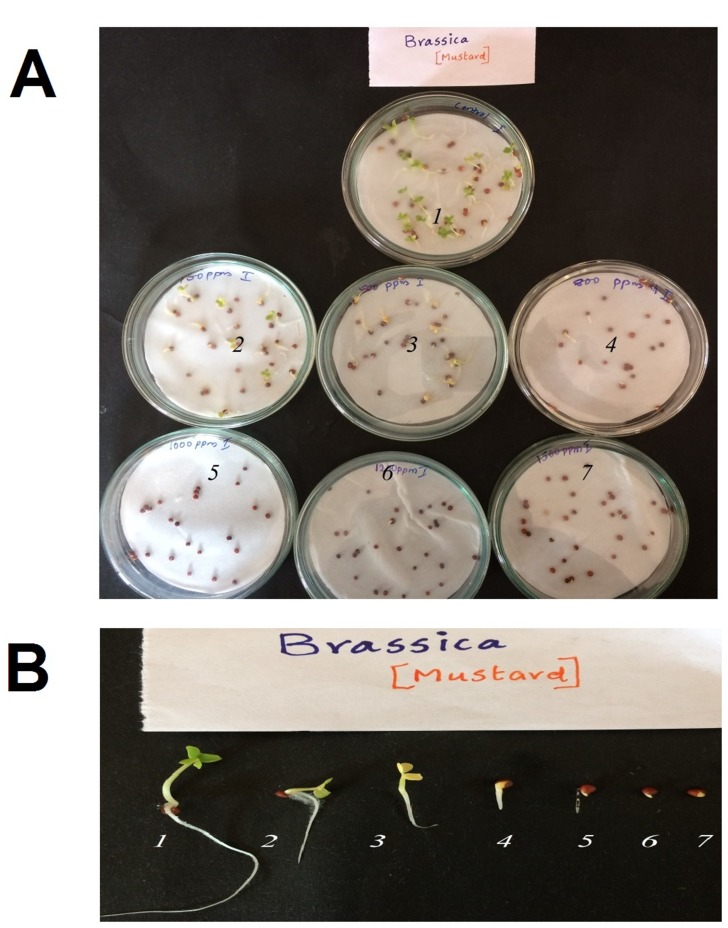
(A) Effect of o-cresol on germination of mustard seeds (Petri plates 1, 2, 3, 4, 5, 6, and 7 contained 0, 100, 200, 800, 1000, 1200 and 1500 mg/l of o-cresol, respectively). (B) Representative seeds (from left to right) germinated in Petri Plates containing 0, 100, 200, 800, 1000, 1200 and 1500mg/l of o-cresol).

All the 13 seeds tested exhibited varying response to o-cresol. Mustard (Brassica juncea) was the most sensitive among them. Marked reduction in germination percentage and remarkable reduction in seedling vigour was observed in these cases, as compared to the control seeds unexposed to o-cresol (Figure 1). There was no germination at all at and above 500 mg/l of the chemical. Detailed results of the filter paper result are shown in Table 1. All other seeds except mustard and okra were found to be either more sensitive or resistant to o-cresol.

Mustard seeds have been highly prized culinary oil-seeds being in use since earlier times. The seeds are made of quality proteins, essential oils, vitamins, minerals, and dietary fiber. Mustard is also known for being high in antioxidants and is also a good source of calcium and potassium. As seed of mustard was found to be very sensitive to o-cresol and also being cultivated all over the world as an important vegetable crop this was selected for detailed studies under soil conditions [10].

#### Soil method

As mustard (Brassica juncea) was found highly sensitive to ocresol and it was selected for a detailed study in soil method.

**Figure 2:**
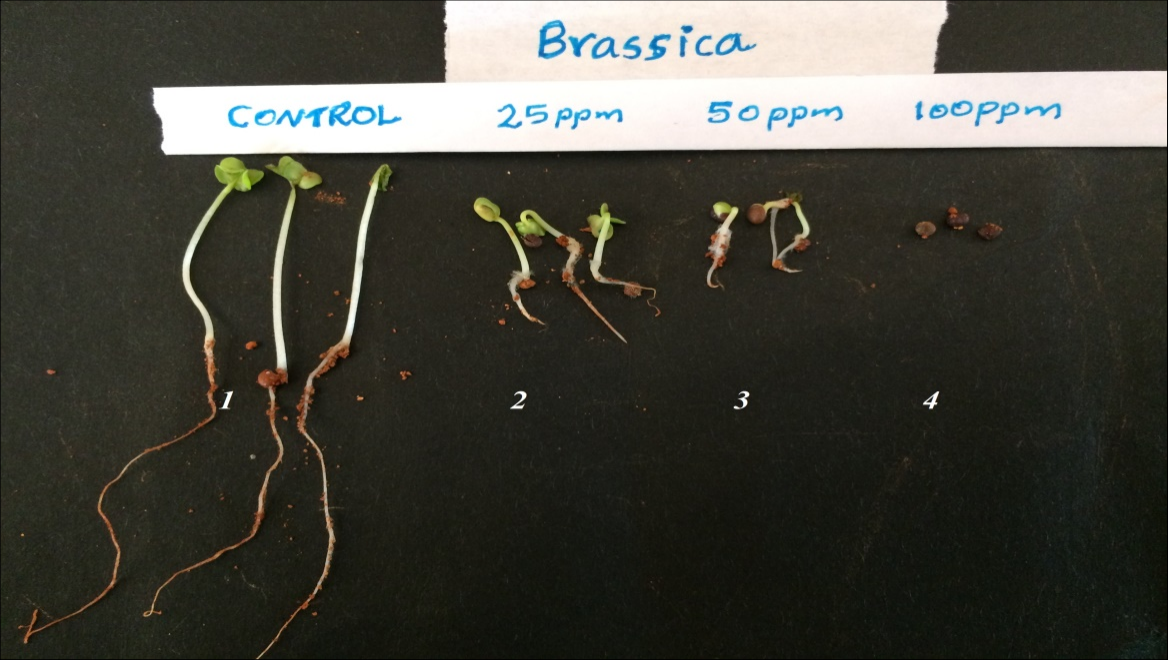
Representative seedlings (after 7 days of growth) of mustard (from left to right) from cups containing 0, 25, 50, and 100mg/kg soil, respectively.

The percentage germination of mustard seeds, their mean shoot and root lengths, and the VI of the seedlings when germinated in cups containing soil spiked with 0, 25, and 50mg/kg o-cresolare given in Table 2. In the control cup all the 25 seeds germinated giving a 100% Germination Percentage. The vigour index was also very high. The germination percentage and the seedling vigour of the mustard seeds in the soil deceased as the concentration of o-cresol increased from 0mg/kg to 25 and 50mg/kg (Figure 2 and Table 2). As there was no germination in cups containing 100, 150 and 200mg o-cresol/kg soil the data are not included in the table.

Germ. (%): Germination percentage; MSL with SD: Mean Shoot length with Standard Deviation. MRL with SD: Mean Root length with Standard deviation; VI: Vigour Index.

Yukiko et al. (2001) [17] have shown that nine phenolic compounds, which were reported as the allelochemicals found in the soil beneath the trees of genus Quercus inhibited the seed germination of shirakamba birch, Betula platyphylla Sukatchev var. japonica Kara. Inhibition of germination of tomato and chicory seeds by olive mill wastewater and pretreatments to remove its toxicity has been reported elsewhere [18, 19] reported a reduced germination rate and radicle growth of tomato and lettuce by pine tree substrate extracts. In the present study also it was observed that root was getting affected more drastically even at a concentration as low as 50mg m-cresol/kg soil. In the case of phenol it was the shoot growth that was getting affected than the root (Amrutha, unpublished data 2014). Ajithkumar et al. (1998) [11] have reported that among the various crop seeds tested the seeds of Solanaceae members such as tomato, eggplant, and tobacco were more susceptible to 3-CBA and 4-CBA.In the case of phenolit was the seeds of chickpea, green gram, and long-podded cowpea, all belonging to the same family, Fabaceae are more susceptible. Gangadhara and Kunhi (2000) [20] also have demonstrated such a phenomenon. They have reported that 2,4,5- T was inhibitory to a number of seeds of eggplant and tomato seeds (both belonging to Solanaceae family) being highly susceptible.

### Viability of mustard seeds exposed to o-cresol in soil

**Figure 3:**
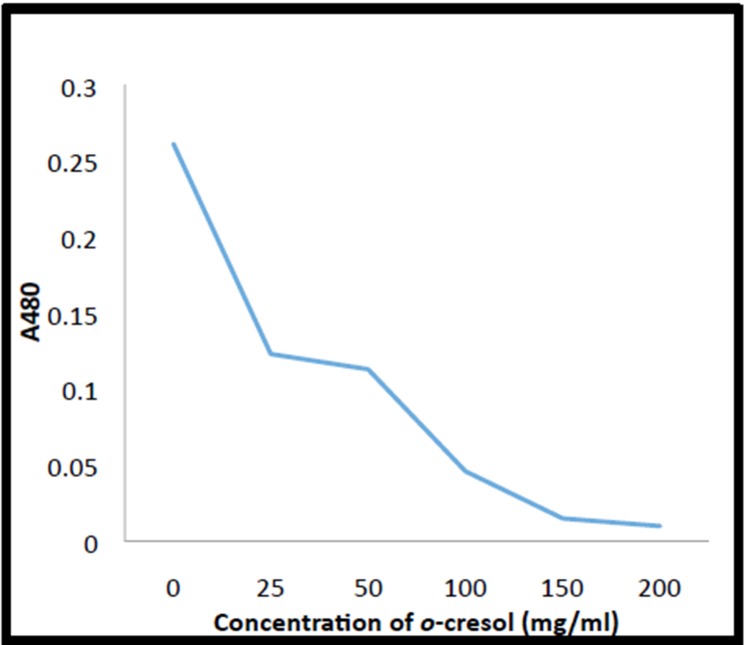
Viability of seeds of B. juncea exposed to different concentrations of o-cresol.

The test indicated a proportionate decrease in viability of mustard seeds with increasing concentration of o-cresol used.The viability loss of mustard seeds was noticed even when 25 mg/l of o-cresol was applied. Actively respiring control seeds showed an OD480 of 0.261. A gradual and consistent decline of seed viability was observed as the concentration of o-cresol was increased and the viability was almost zero in seeds exposed to 200 mg/l of the chemical (Figure 3). It could be inferred that exposure to o-cresol inhibits the activity of dehydrogenases that catalyze mitochondrial respiration, thus rendering the seeds non-viable.

Similar observations have been made by Ajithkumar et al., (1998), Gangadhara and Kunhi A. A. M. (2000), and Amrutha Vijay (2014) [11, 20] in the case of tomato seeds when exposed to different concentrations of chlorobenzoates (3-CBA/4-CBA), 2,4,5-T and phenol, respectively. According to Peterson et al., (1996) [21] tall fescue seeds exposed to TNT and 4ADNT also showed loss of viability. This proves that the reduction or complete elimination of germination at different concentrations of the chemicals is due to the failure of the respiratory mechanism of the cells. Recently, Devkota and Jha (2010) [22] based on their studies stated that viability of seeds deteriorated as duration of storage increased and they became non-viable after storing it for thirty months.

### Studies of Seed-Bourne Enzymes: Protease Activity

#### Protease Activity

**Figure 4:**
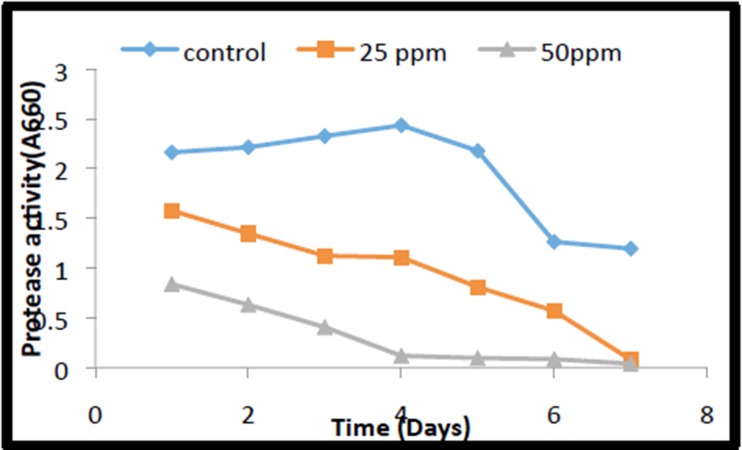
Protease activity of mustard seeds exposed to different concentrations of o-cresol at different period of germination.

Generally, storage proteins present in the seeds are degraded by activation of protease enzymes during seed germination to provide nutrients to embryo and seedling growth [23, 24] In the control seeds the protease activity showed a steady increase till day after sowing and remained almost content till 4th day and after that started declining (Figure 4). But, in the case of seeds germinating in soil containing 25 and 50 mg/kg soil, the protease activity continuously declined till the day7. The reduction in protease activity in this case of 50mg/kg soil was more drastic than that was in 25 mg/kg grown seeds.

#### Amylase Activity

**Figure 5:**
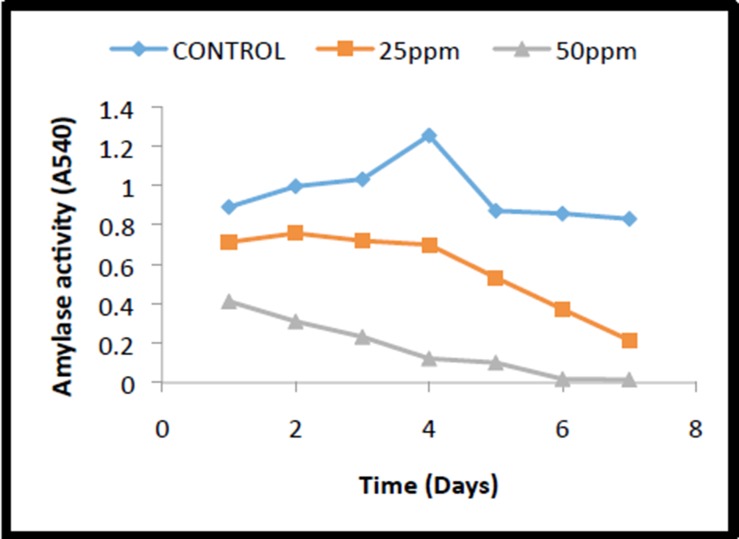
Amylase activity of mustard seeds exposed to different concentrations of o-cresol at different periods of germination.

In the present study, the increased activity for total amylase was observed in the initial days of germination of control seeds until fourth day and then started declining gradually as depicted in the Figure 5. However, in the case of seeds exposed to o-cresol the activity was drastically reduced from the beginning itself, which steeply came down and reached zero level on the day 7 (Figure 5). Similar kind of results for amylase activity was observed in Zea mays by Sangeetha [2013] [25] and these results were in accordance with the results of present study. Amylase play a major role in carbohydrate metabolism in several plant tissues and starch is the major component of most of the world's crop yield and the degradation of starch is essential in the germination of these plants [26]. An increase in amylase, protease and other enzymatic activities have been reported by several workers in the seeds of legumes viz. mung bean, lentil, cowpea, chickpea, pea, horse gram, moth bean, and field bean during normal germination reaching a maximum within 3 to 4 days [27, 32].

In the present study also, it was observed that protease enzymes were getting activated in control seeds un-exposed to o-cresol. The results of protease and amylase activities in seeds of mustard exposed and un-exposed to o-cresol obtained in the present study were in agreement with the observations made by other workers with other chemicals.

### Effect of inoculation of o-cresol-spiked soil with *Pseudomonas monteilii* SHY on seed germination and seedling vigour of mustard:

BI: Bacterial inoculum; Germ. (%): Germination percentage; MSL with SD: Mean Shoot length with Standard Deviation. MRL with SD: Mean Root length with Standard deviation; VI: Vigour Index.

**Figure 6:**
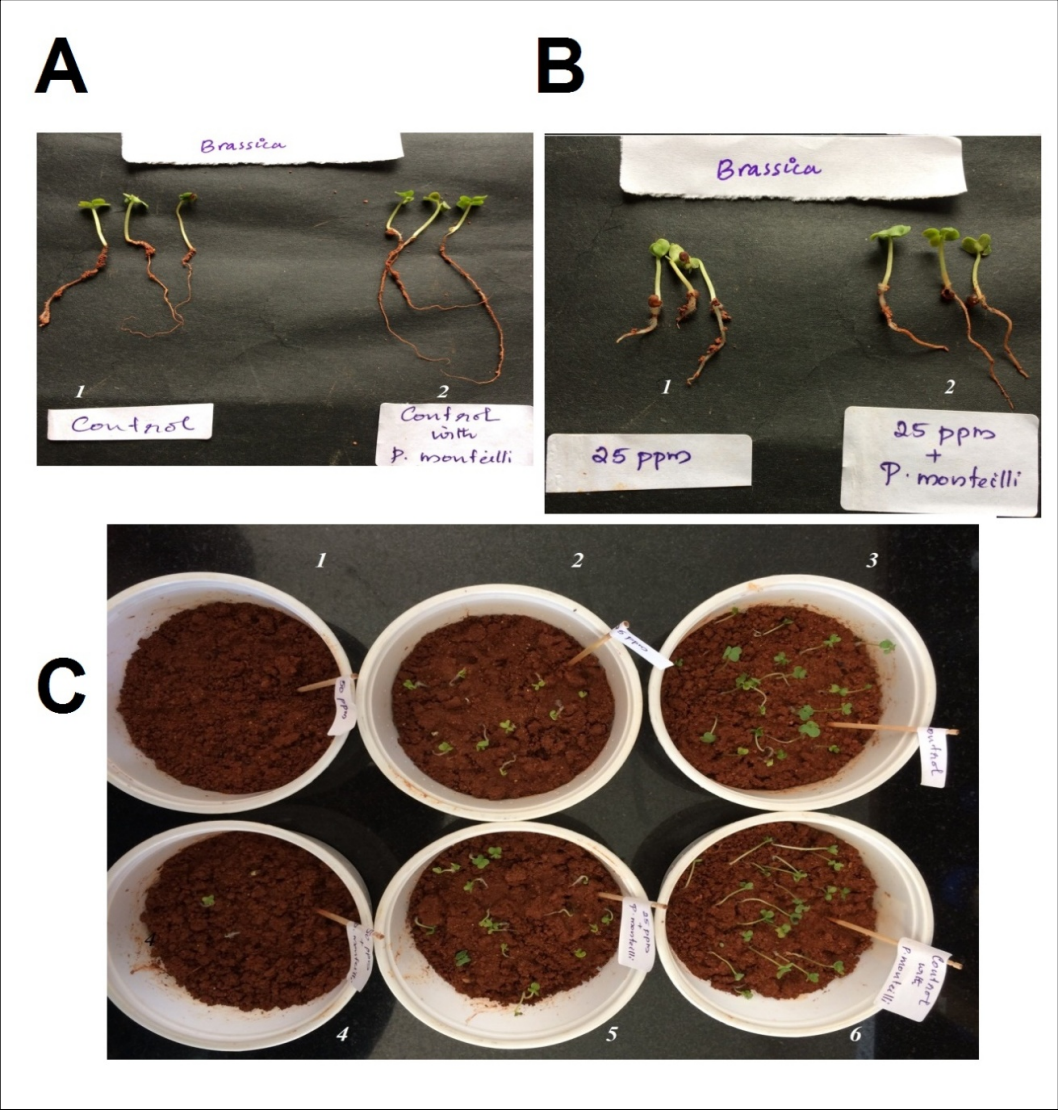
(A) Representative plants; (1) Control Soil with no chemical; (2) Soil with bacterium-inoculated; (B) representative plants; (1) Seeds grown in 25 mg of o-cresol without BI (2) seeds grown in 25mg of o-cresol with BI; (C) Effect of different concentrations of ocresol on germination of mustard seeds (plastic cups1, 2, 3, 4, 5 and 6) UI-un inoculated: BI-Bacterial Inoculation. (3) Control - Soil with no chemical and no BI, (4)-25 + UI-Soil with 25 mg o-cresol/kg, (5)- 25+BI - Soil with bacterium-inoculated and 25 mg o-cresol (1)- 50 + un inoculated and(2) 50 + BI - Soil with 50 o-cresol/kg, (6)-Control + BI - Soil with bacterium-inoculated without chemical.

Complete recovery of germination percentage and seedling vigour of mustard seeds were obtained after the inoculation of the soil with the bacterial cells proving the efficacy of P. monteilii SHY in bio-remediating the soil from o-cresol (Figure 6A and 6B). In the case of 25mg/kg soil of o-cresol the germination percentage increased to 64% from 36% that of BI un-inoculated cups and the VI increased from 111 to 396 that of BI uninoculated cups. The germination percentage of both inoculated and un-inoculated control seeds not exposed to the chemical was 88% and 96% respectively and the VI was 669 and 456, respectively. In the case of cups with 50 mg o-cresol/kg soils the % germination improved from 0 to 20% when inoculated with bacterium. The VI shot up from 0 to 68 (Table 3). Probably with a little higher inoculum size the recovery could be complete, which, of course, need to be experimentally verified. Slight improvement in the seedling vigour was observed in the bacteriainoculated cups without the chemicals. It is possible that some of the intermediary metabolites may be having plant growth promoting effect. However, this needs to be experimentally verified. Bioremediation has been established as an effective method of elimination of toxic chemicals from polluted sites [33, 35]. One of the strategies adopted has been bioremediation through cell augmentation or gene augmentation using microorganisms possessing degrading potentials, and a few studies have indicated the possibility of successful application of such processes [11, 20].

In a similar study Gangadhara and Kunhi (2000) [20] have shown that bioremediation of the 2, 4, 5-T-contaminated soil by inoculation with Burkholderia cepacia AC1100 completely protected the germination of tomato seeds. Bidlan et al. (2004) [16] have reported bioremediation of HCH-contaminated soil by a mixed microbial culture and elimination of the inhibitory effects of the insecticide on seed germination of radish and green gram. Recently, Amrutha, (unpublished data, 2014) have shown elimination of the inhibitory effect of phenol on chickpea seed germination through bioremediation of the soil with strain of P. aeruginosa S-CSR 0013. However, the complete recovery from the inhibitory effect of phenol was possible only if the seeds were sown 8 days after the bacterial inoculation. Similarly, Krueger et al. (1991) [35] have shown protection of soybean and pea seedlings from the deleterious effects of the herbicide, dicamba by inoculating soils with dicamba-degrading bacteria.

### Growth of bacterial strain in o-cresol-spiked and un-spiked soil:

**Figure 7:**
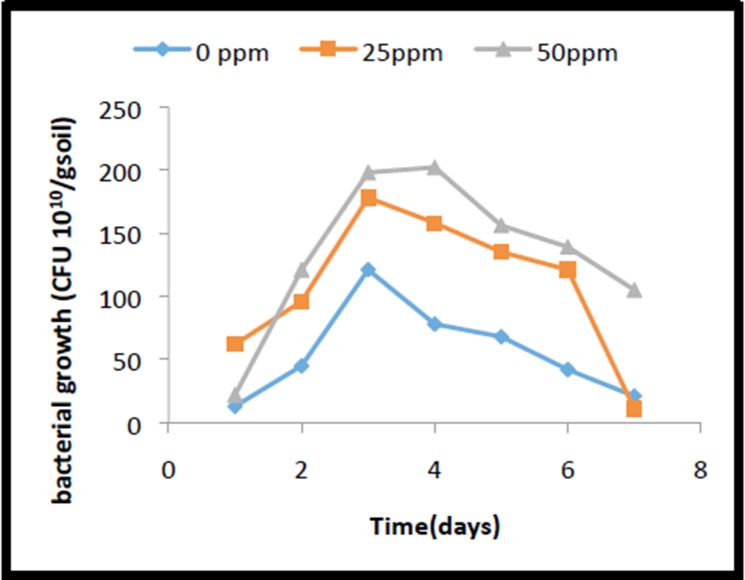
Bacterial growth in soil when different concentrations of o-cresol was provided.

Viable count of the bacterial strain P. monteilii SHY was made and extreme care was taken to exclude the contaminated bacteria by way of using autoclaved soil for germination studies as well as using selective media (M3) media with cresol as substrate for plate counts. In case of both cresol-spiked and un-spiked control soil, the cells of P. monteilii SHY were found to be viable and actively growing as shown in the Figure 7. There was a slight lag of 2 days before the active growth of the bacterium began. Then there was a steep increase in growth, which reached the peak on 5th day in the case of 25 mg o-cresol/kg soil. Then there was a fast death phase.

Ajithkumar et al., (1998) and Gangadhara and Kunhi (2000) [11, 20] have reported the survival and growth of P. aeruginosa 3mT, B. cepacia AC1100, and P. aeruginosa S-CSR-0013 in soil containing 3-CBA/4-CBA, 2,4,5-T, and phenol, respectively. The bacterium also exhibited a little growth in the control soil without o-cresol, probably by utilizing any little nutrient available therein.

### Degradation of o-cresol in soil by *Pseudomonas monteilii* SHY

**Figure 8:**
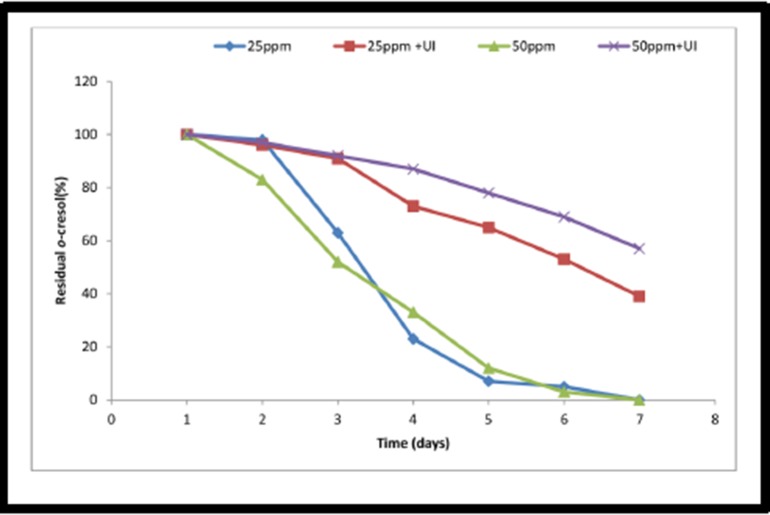
Degradation of different concentrations of o-cresol in soil inoculated with Pseudomonas monteilii SHY.

The o-cresol degrading efficiency of the inoculated bacterium was monitored through the estimation of the residual substrate in soil. There was a fast degradation of the added chemical in the bacterium-inoculated soil (Figure 8). Within 4 days the level of both 25 and 50mg/kg of o-cresol was brought down to less than 10%, which was then taken to almost zero on day 7. In the uninoculated soil the chemical persisted for a long period. The disappearance of o-cresol from the un-inoculated soil was very slow. Even after 8 days of incubation, almost 60 and 70% (i.e. about 30 and 105mg/kg soil) of the added o-cresol was present. Faster growth of the inoculated organism with concomitant degradation could be the reason why the seeds were protected, although not completely, even when they were sown immediately. (Key: 25ppm - 25mg o-cresol/kg soil inoculated with bacterium, 25ppm +UI - 25mg o-cresol/kg soil uninoculated; 50mg o-cresol/kg soil inoculated with bacterium, 50ppm +UI - 50mg o-cresol/kg soil un-inoculated). In a similar study, Ajithkumar et al. (1998) [11] have reported that in the case of chlorobenzoate-spiked soil where the inoculated P. aeruginosa 3mT grew fast and eliminated the inhibitory effect of the chemicals enabling the normal germination of tomato seeds.

## Conclusion

We tested 13 different vegetable seeds for o-cresol sensitivity. Mustard and okra were found to be highly vulnerable to o-cresol. Even low concentrations of o-cresol partially or completely inhibited the seed germination and drastically reduced the seedling vigour. It has also been shown that these harmful effects can effectively be eliminated by bioremediation of o-cresolcontaminated soil by inoculating with P. monteilii SHY. However, the data presented here pertain to laboratory studies, and detailed field trials have to be carried out to validate the findings and to ascertain the suitability of this bioremediation technique under natural conditions.

## Figures and Tables

**Table 1:** Preliminary screening data by filter paper method

Seeds	Days	Concentrations of o- cresol							comment
		Control	100ppm	500ppm	800ppm	1000ppm	1200ppm	1500ppm	
Green gram	1	yes	yes	yes	yes	yes	yes	yes	Rejected, No inhibition
	II	yes	yes	yes	yes	yes	yes	yes	
Chick pea	1	yes	yes	yes	yes	yes	yes	yes	Rejected, No inhibition
	II	yes	yes	yes	yes	yes	yes	yes	
Green pea	1	yes	yes	yes	yes	yes	yes	No	Rejected, No inhibition
	II	yes	yes	yes	yes	yes	yes	yes	
okra	1	yes	yes	yes	No	No	No	No	SELECTED
	II	yes	yes	yes	No	No	No	No	
Red chilli	1	No	No	No	No	No	No	No	Rejected very high germination time
	II	No	No	No	No	No	No	No	
Brinjal	1	No	No	No	No	No	No	No	Rejected very high germination time
	II	No	No	No	No	No	No	No	
Tomato	1	No	No	No	No	No	No	No	Rejected very high germination time
	II	No	No	No	No	No	No	No	
Fenugreek	1	yes	yes	yes	yes	No	No	No	Rejected, No inhibition
	II	yes	yes	yes	yes	yes	yes	yes	
Mustard	1	yes	yes	No	No	No	No	No	SELECTED
	II	yes	yes	yes	No	No	No	No	
Cow pea	1	yes	yes	yes	yes	yes	yes	yes	Rejected, No inhibition
	II	yes	yes	yes	yes	yes	yes	yes	
Coriander	1	No	No	No	No	No	No	No	Rejected very high germination time
	II	No	No	No	No	No	No	No	
Spinach	1	No	No	No	No	No	No	No	Rejected, due to its small size
	II	yes	No	No	No	No	No	No	
Cucumber	1	yes	yes	yes	yes	No	No	No	Rejected, No inhibition
	II	yes	yes	yes	yes	yes	yes	yes	

**Table 2:** Effect of o-cresol on germination and seedling vigour of mustard (Brassica juncea) seeds as tested in soil.

O-C/ Conc mg/L	G%	MSL SD (cm)	MRL SD (cm)	VI
0ppm(Control)	100	2.14 + 0.52	4.324 + .99	646.4
25ppm	64	1.04 + 0.02	1.47 + 0.2	160.64
50ppm	12	0.3 + 0	0.57 + .01	10.44

**Table 3:** Effect of amendment of soil with Pseudomonas monteilii SHY cells on germination and seedling vigour of mustard seeds.

Concentration of o-cresol (mg/kg soil) with or without BI.	Germ. (%)	MSL with SD (cm)	MRL with SD (cm)	Vigour Index
Control	88%	1.99±0.2	3.20±0.76	456.72
Control + BI	96%	1.95±0.58	5.02±1.3	669.12
25	36%	1.16±0.2	1.94±0.24	111.6
25+BI	64%	1.4±0.1	4.8±1.5	396.8
50	0	0	0	0
50+BI	20%	1.21±0.4	2.2±.5	68
